# Novel Blood–Brain Barrier Shuttle Peptides Discovered through the Phage Display Method

**DOI:** 10.3390/molecules25040874

**Published:** 2020-02-17

**Authors:** Petra Majerova, Jozef Hanes, Dominika Olesova, Jakub Sinsky, Emil Pilipcinec, Andrej Kovac

**Affiliations:** 1 Institute of Neuroimmunology, Slovak Academy of Sciences, Dubravska cesta 9, 845 10 Bratislava, Slovakia; petra.majerova@savba.sk (P.M.); jozef.hanes@savba.sk (J.H.); dominika.olesova@savba.sk (D.O.); jakub.sinsky@savba.sk (J.S.); 2 Department of Microbiology and Immunology, The University of Veterinary Medicine and Pharmacy, Komenskeho 73, 04181 Kosice, Slovakia; emil.pilipcinec@uvlf.sk; 3 Department of Pharmacology and Toxicology, The University of Veterinary Medicine and Pharmacy, Komenskeho 73, 04181 Kosice, Slovakia

**Keywords:** blood–brain barrier, phage display, shuttle peptides

## Abstract

Delivery of therapeutic agents into the brain is a major challenge in central nervous system drug development. The blood–brain barrier (BBB) prevents access of biotherapeutics to their targets in the central nervous system and, therefore, prohibits the effective treatment of many neurological disorders. To find blood–brain barrier shuttle peptides that could target therapeutics to the brain, we applied a phage display technology on a primary endothelial rat cellular model. Two identified peptides from a 12 mer phage library, GLHTSATNLYLH and VAARTGEIYVPW, were selected and their permeability was validated using the in vitro BBB model. The permeability of peptides through the BBB was measured by ultra-performance liquid chromatography-tandem mass spectrometry coupled to a triple-quadrupole mass spectrometer (UHPLC-MS/MS). We showed higher permeability for both peptides compared to N–C reversed-sequence peptides through in vitro BBB: for peptide GLHTSATNLYLH 3.3 × 10^−7^ cm/s and for peptide VAARTGEIYVPW 1.5 × 10^−6^ cm/s. The results indicate that the peptides identified by the in vitro phage display technology could serve as transporters for the administration of biopharmaceuticals into the brain. Our results also demonstrated the importance of proper BBB model for the discovery of shuttle peptides through phage display libraries.

## 1. Introduction

The blood–brain barrier (BBB) represents the bottleneck in brain drug development and is the most critical factor limiting the future growth of neurotherapeutics. The BBB is a selective semi-permeable barrier formed by the endothelial cells that shape cerebral microvessels and separate blood from the brain. Therapeutic strategies to deliver drugs into the central nervous system (CNS) are limited by the restrictive tight junctions among the endothelial cells of BBB. Ideal drug candidates are small, lipophilic, hydrophobic, and compact molecules that can easily cross the BBB [1]. Large molecules, for example, peptides, recombinant proteins, monoclonal antibodies, RNA interference (RNAi)-based drugs, and >98% of small-molecule drugs do not cross the BBB [2]. Generally, peptides cross the BBB through endocytic mechanisms involving receptor-mediated transcytosis (RMT) and/or adsorptive-mediated transcytosis (AMT) [3], whereby both belong to active mechanisms of transport. In the process of transcytosis, the formed vesicles circulate across the cell, bypassing the degradation pathway. Eventually, they can release their content into the parenchyma (exocytosis). Adsorptive-mediated transcytosis is considered non-specific and comprises all vesicular transport mechanisms that do not involve protein receptors [4]. In AMT, endocytosis is often promoted by the interaction of the positively charged molecule with membrane phospholipids. Adsorptive-mediated transcytosis provides the highest transport since the first is considered unsaturable and saturation concentrations for AMT are higher than for RMT.

To bypass the BBB and to deliver therapeutics into the brain, several CNS delivery strategies have been developed [5]. The most promising approach among them is receptor-mediated transport (RMT). Receptor-mediated transport can be used for the transport of large molecules like proteins or antibodies through BBB. This strategy is based on the conjugation of the biotherapeutic agent with a “vector” which is part of a natural ligand or peptide and is recognized explicitly by a receptor on brain endothelial cells. After interaction with the receptor, a conjugate is transported through the BBB via endocytosis. In this way, biotherapeutics can cross the endothelium and enter the brain without disruption of barrier properties. Several RMT transporting systems have been described, for example, transferrin receptor [6,7,8,9], insulin receptor [10,11,12], insulin-like growth factor receptor [13,14,15], low-density lipoprotein receptor-related protein 1 [16], low-density lipoprotein receptor-related protein 2 [17,18], and diphtheria toxin receptor [19,20].

Few reports have described BBB shuttle peptides that do not require receptors as targeting vectors for BBB delivery [21,22,23]. Cell-penetrating peptides (CPPs) and peptides undergoing passive diffusion across the BBB do not provide brain selectivity. However, their internalizing capacity can be fine-tuned or exploited in tandem with BBB shuttles to enhance the delivery of cargoes to the brain. Although several studies have documented a fast bulk brain accumulation, only a few of them showed an improved therapeutic effect [24,25,26]. Cell-penetrating peptides act as cargo carriers and constitute a current hotspot in medical research. They are capable of entering the tissues in a non-invasive manner and accelerate the absorption of macromolecules via physiological mechanisms such as energy-dependent endocytosis and energy-independent direct penetration. Cell-penetrating peptides can be refolded and assembled with synthetic nanostructures to improve the disadvantages owing to non-selective, lower delivery efficiency, and decreased susceptibility to degradation. Cell-penetrating peptides can also be incorporated into cargo-carrying platforms to create novel drug-delivery systems that ensure improved coated-drug uptake and controlled release via stimulus-responsive mechanisms [27,28,29,30,31]. The use of CPPs in drug delivery could improve the potency of protein- or nucleic-acid-based agents by increasing the possibility of drugs to cross physiological barriers such as BBB, nose mucous membrane, gastrointestinal mucosa or skin [27].

Several CPP peptides have been extensively studied. For delivery of small molecules (<300 Da), short 2–4 amino acid peptides, such as diketopiperazines, methylphenylalanines, and phenylprolines, have been developed [22,23,32,33]. The TAT peptide (GRKKRRQRRRPQ) is the best-studied CPP for brain delivery for proteins and nanoparticles. It was demonstrated that TAT peptide-coated nanoparticles were efficiently internalized into brain capillary endothelial cells by adsorptive-mediated endocytosis [31,34,35,36]. The most clinically advanced CPP technology for BBB transport uses Angiopep-2 peptide developed by Angiochem Inc. (Montreal, QC, Canada) [37,38]. The company has started clinical development with its most advanced product, ANG1005 (paclitaxel conjugated to Angiopep-2). Two clinical phases I studies using ANG1005 have already been completed (first study in patients with malignant glioma, second study in patients with advanced solid tumors and metastatic brain cancer). Another delivery peptide, designated COG133, is an apoE-mimetic peptide derived from amino acids 133–149 of ApoE. It retains its biological activity in vitro and in vivo [39,40,41], and this region of ApoE is critical for interaction with the low-density lipoprotein (LDL) receptor. A peptide designated as TGN was also transported efficiently into the brain. The TGN peptide decorated nanoparticles loaded with fluorescent cumarine 6 showed accumulation in the brain, whereby the accumulation increased with a higher number of peptides conjugated to nanoparticles [25,42]. An in vivo phage display approach was used for in vivo identification of the clone7 peptide from cyclic 7-mer phage display library C7C using laboratory rats [43]. Phage presenting clone7 peptide exhibited about 50-fold higher translocation efficacy to rat brain compared to a random phage. An 11 amino acid synthetic peptide derived from the displayed sequence of clone7 applied intranasally in rats bypassed the BBB and entered the brain directly [44].

In the present work, we used phage display technology to find new BBB shuttle peptides. We identified peptides that bound specifically to primary endothelial cells and could traverse the BBB.

## 2. Results

### 2.1. Identification of Peptides Specifically Binding to Primary Rat Endothelial Cells

**Figure 1:**
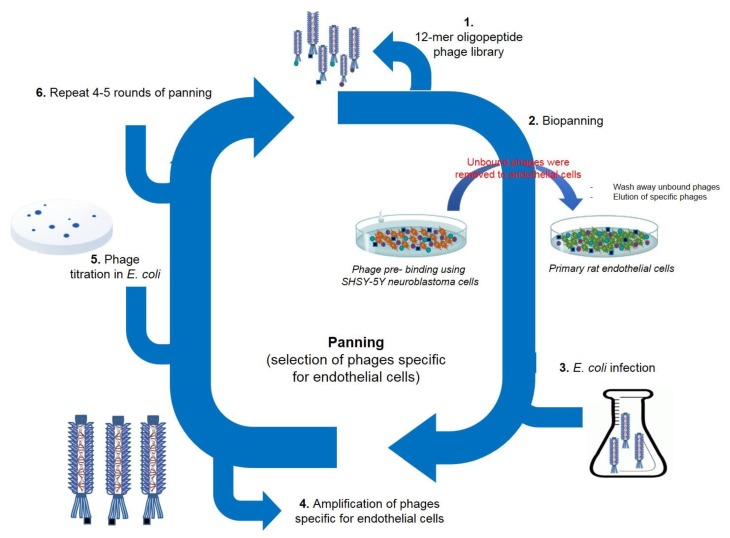
Bio-panning experiments. Selection of phages specific for endothelial cells. Ph.D.-12^TM^ Phage Display Peptide Library was first incubated with neuroblastoma cells to exclude promiscuous binding peptides. The non-bound phages were then screened on primary rat brain endothelial cells. We performed four rounds of bio-panning.

**Figure 2:**
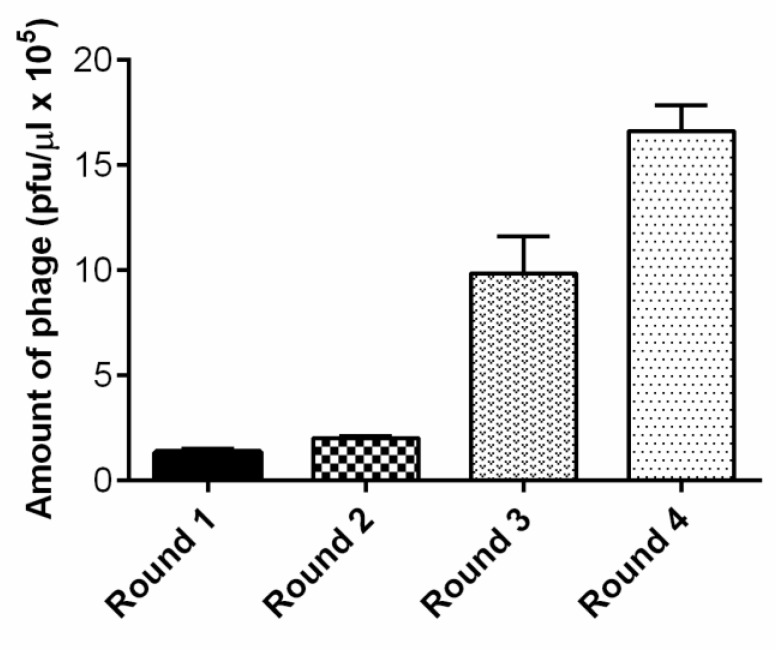
The number of phages recovered from endothelial cells increased after each round of bio-panning.

To perform the panning against primary rat endothelial cells, the Ph.D.-12^TM^ Phage Display Peptide Library was selected as the initial phage pool. To exclude promiscuous binding phages in the screening, we first performed phage panning experiments on neuroblastoma SH-SY5Y cells. The non-bound phages were then screened on primary rat brain endothelial cells. We conducted four rounds of bio-panning (Figure 1). The number of phages, specifically recognizing endothelial cells, increased after each round of panning, and the number of recovered phages after the fourth round increased 12 fold compared to the first round (Figure 2). After the first round, we detected 1.3 × 10^5^ pfu/µL; after the second round 2 × 10^5^ pfu/µL. After the third round, we detected a markedly higher number of phages: 9.8 × 10^5^ pfu/µL. After the fourth and last round, we obtained 1.6 × 10^6^ pfu/µL.

After the fourth round, the DNA of individual phages was isolated. A total of 35 phage clones were chosen randomly from the final round of bio-panning and subjected to DNA sequencing. We identified 14 different peptides, whereby two of them were found more than three times: GLHTSATNLYLH (11×) and VAARTGEIYVPW (8×). We selected these two most abundant peptides for further validation as candidates for a new BBB shuttle. The sequences of the identified peptides are shown in Table 1. Calculated physico-chemical properties are shown in Table S1. For further experiments we used N-C reversed sequence peptides: HLYLNTASTHLG and WPVYIEGTRAAV as the control.

### 2.2. High-Resolution Mass Spectrometry Analysis and LC–MS/MS Analysis

**Figure 3:**
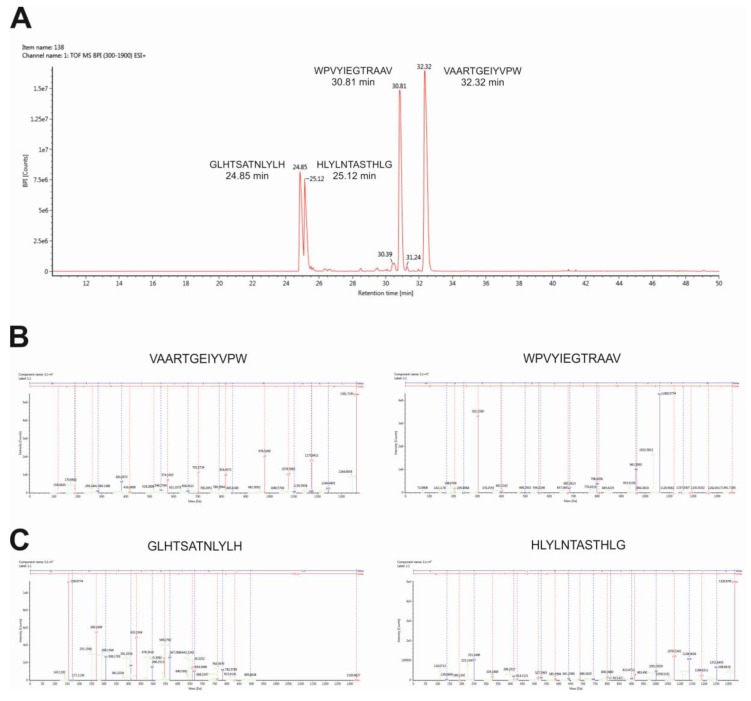
Identification of peptides by high-resolution mass spectrometry analysis. Total ion chromatogram (**A**), fragmentation spectra of N–C reversed sequence peptide WPVYIEGTRAAV and VAARTGEIYVPW (**B**), fragmentation spectra of peptide GLHTSATNLYLH, and peptide N-C reversed sequence peptide HLYLNTASTHLG (**C**) are shown.

To verify the identity of peptides and to characterize fragments, we performed high-resolution mass spectrometry analysis using the Synapt-G2Si instrument. As shown in Figure 3A, the total ion chromatogram showed four major peaks at retention times from 24–34 min. The main peaks in the total ion current (TIC) spectrum were identified as predicted peptide GLHTSATNLYLH (24.85 min, *m/z* 965.5020), peptide HLYLNTASTHLG (25.12 min, *m/z* 965.5020), peptide WPVYIEGTRAAV (30.81 min, *m/z* 965.5020), and peptide VAARTGEIYVPW (32.32 min, *m/z* 965.5020). The MS/MS fragmentation spectra clearly confirmed the identity of the transported peptides (Figure 3B,C).

**Figure 4:**
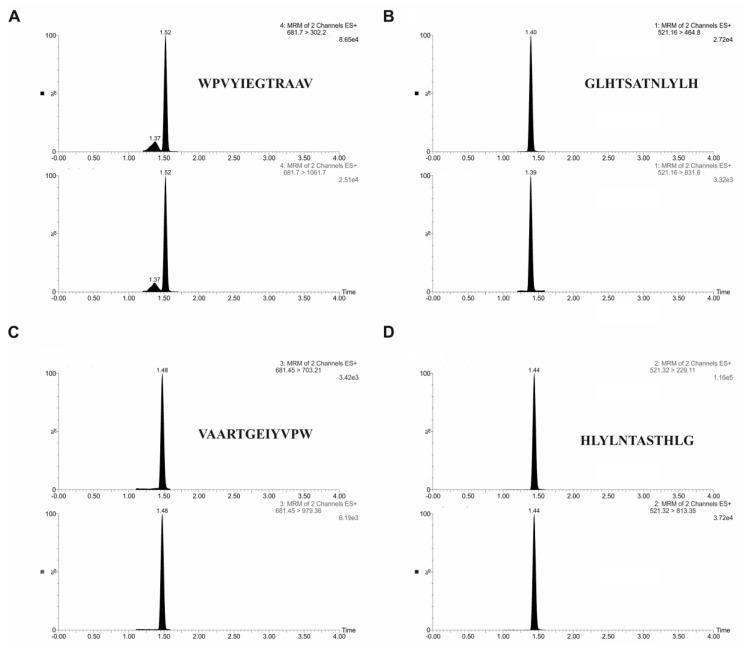
Selected reaction monitoring (SRM) chromatograms of peptides: (**A**) WPVYIEGTRAAV; (**B**) GLHTSATNLYLH; (**C**) VAARTGEIYVPW; and (**D**) HLYLNTASTHLG.

Quantitative analysis of peptides was carried out by ultra-performance liquid chromatography-tandem mass spectrometry using ACQUITY ultra-performance liquid chromatography (UPLC) I-class system coupled to a triple-quadrupole mass spectrometer Xevo TQD. Two characteristic fragments for each peptide were selected for quantification experiments. For the optimal separation, the gradient elution program was established, using water with formic acid and acetonitrile. Table 2 demonstrates the mass spectrometry parameters used for analysis. The retention time for targeted analytes was 1.40 min for VAARTGEIYVPW peptide, 1.44 min for WPVYIEGTRAAV peptide, 1.52 min for GLHTSATNLYLH peptide, and 1.48 min for HLYLNTASTHLG peptide. Figure 4 displays a typical chromatogram of targeted analytes in standard solution.

### 2.3. Endothelial Cytotoxicity and Permeability of Peptides across In Vitro Blood–Brain Barrier (BBB) Model

The peptide cytotoxicity was determined by the measurement of the levels of adenylate kinase in the culture supernatants. There was no statistically significant increase of adenylate kinase levels up to 10 µM concentration (Figure S1).

The permeability of peptides was assessed using a primary rat in vitro BBB model. Different qualitative and quantitative techniques were used to confirm BBB integrity. Transendothelial electrical resistance (TEER) measurements were used to measure the resistance of tight junctions in our BBB model. For our experiments, we used only inserts with the TEER value ≥320 ± 20 Ώ cm^−2^ that corresponded to an intact in vitro BBB model. Alternatively, the integrity of the in vitro rat BBB model was assessed by measuring the permeability of the paracellular compound Lucifer Yellow (LY). Lucifer Yellow was added to the well with the peptides. The endothelial permeability coefficient (Pe) of LY was around 11 × 10^−6^ cm/s.

To test the permeability of selected peptides across in vitro BBB model, peptide transport to the abluminal compartment was quantified by ultra-high-performance liquid chromatography/mass spectrometry (UHPLC–MS/MS) method.

For this analysis, we selected the two most abundant peptides identified by phage display, peptide GLHTSATNLYLH, and peptide VAARTGEIYVPW. As a negative control, we performed the permeability experiments with N–C reversed sequence peptides: HLYLNTASTHLG and WPVYIEGTRAAV. After the evaluation of the results, we calculated the permeability coefficient for all four peptides. We obtained following permeabilities through in vitro BBB: for peptide GLHTSATNLYLH 3.3 × 10^−7^ cm/s and for peptide VAARTGEIYVPW 1.5 × 10^−6^ cm/s. The permeabilities obtained for N–C reversed sequence peptides were: 7.5 × 10^−7^ cm/s for N–C reversed sequence peptide HLYLNTASTHLG and 5 × 10^−7^ cm/s for N–C reversed sequence peptide WPVYIEGTRAAV. The results from permeability experiments clearly showed that the control N–C reversed sequence peptides were transported across in vitro BBB with less efficiency.

### 2.4. Internalization of Peptides into Endothelial Cells

**Figure 5:**
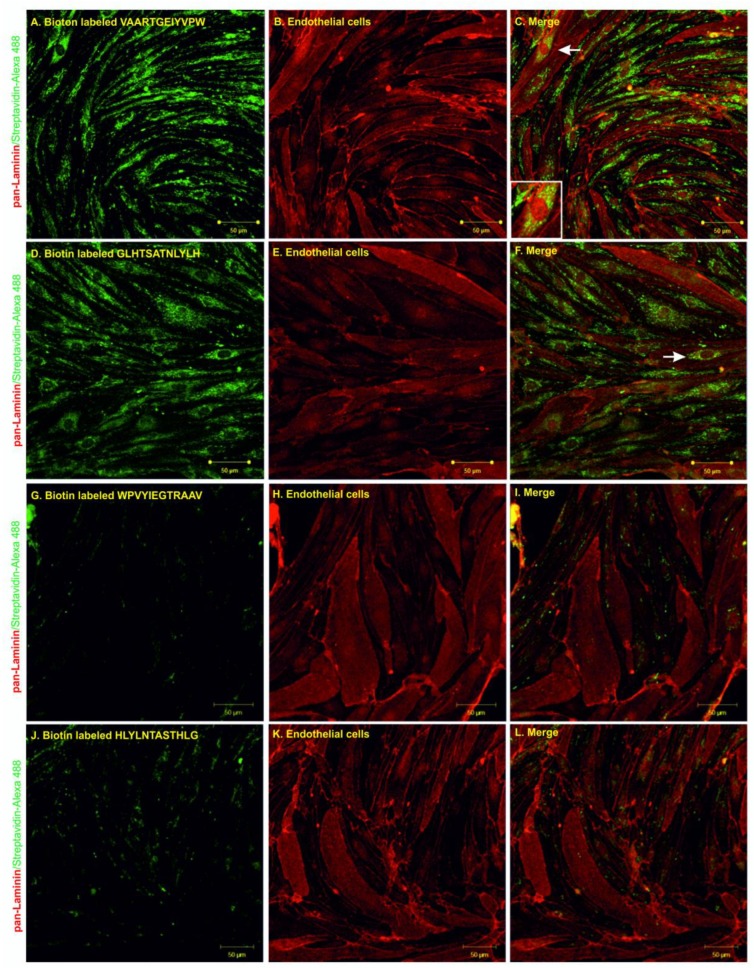
Confocal microscopy of endothelial cells after 1 h of co-incubation with VAARTGEIYVPW (**A**–**C**), GLHTSATNLYLH (**D**–**F**), WPVYIEGTRAAV (**G**–**I**) and HLYLNTASTHLG (**J**–**L**) peptides. Biotinylated peptides are shown in green and pan-laminin signal is shown in red. White arrows indicate examples of the internalization of peptides into endothelial cells. Scale bar = 50 µm.

The internalization experiments were performed by incubating the biotin-labeled peptides with primary rat endothelial cells for 1 h at 37 °C and then evaluating peptide uptake using confocal microscopy. Pan-laminin protein staining was used as a marker for endothelial cells; streptavidin Alexa 488 was used to visualize the biotinylated peptides. The results from confocal microscopy showed that both peptides, GLHTSATNLYLH and VAARTGEIYVPW, were able to internalize into primary endothelial cells (Figure 5A–F). The internalization of N–C reversed sequence peptides into primary endothelial cells was negligible (Figure 5G–L). Additionally, there was no binding or internalization into control non-related cell types—neuroblastoma cells (Figure S2).

**Figure 6:**
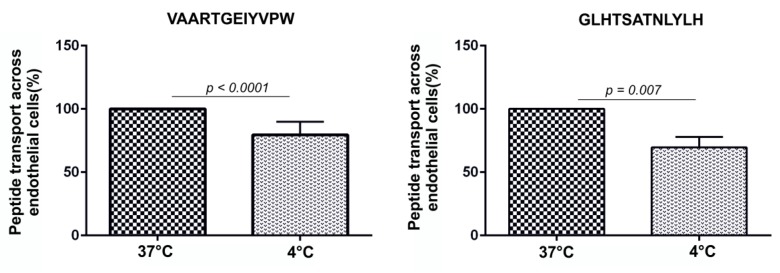
The transport of peptides across the in vitro BBB model was affected by temperature. To test whether the transport of peptides was performed by active uptake, endothelial cells were incubated with peptides at 37 °C and also at 4 °C.

To test the effect of temperature on the transport of peptides across BBB, endothelial cells were incubated with peptides at 4°C to reduce cell metabolism which slows down endocytosis. We observed that lower temperatures affected endocytosis and peptide permeability across the BBB model. The permeability of VAARTGEIYVPW was reduced by 30.5% at 4°C (37 °C: 100%; 4 °C: 69%; *n* = 6; *p* > 0.0001). The permeability of GLHTSATNLYLH was reduced by 20.5% at 4°C (37 °C: 100%; 4 °C: 79%; *n* = 6; *p* > 0.007). This result indicates that the tested peptides were internalized into endothelial cells by an energy-dependent process (Figure 6).

## 3. Discussion

The BBB is a selective barrier, which prevents the entrance of compounds from the blood into the brain [45]. However, in the treatment of neurological diseases, it is necessary to transport therapeutics to their targets which are present behind the BBB. Delivery of therapeutics to the brain is a significant challenge in CNS drug development [46]. Many strategies to circumvent the BBB have been proposed.

In the current study, we used phage display technology to identify peptides that can be effectively transported across BBB. For the peptide screening, we selected the primary rat endothelial cells. To perform the panning against the in vitro primary rat endothelial cells, the Ph.D.-12^TM^ Phage Display Peptide Library was selected as the initial phage pool. Using an in vitro screening approach, we identified phages which specifically bound to the endothelial cells. From the panning experiment, we selected and analyzed 35 phages. Among them, we identified phages displaying all together 14 different peptides, whereby two of them were found with the highest occurring frequency: VAARTGEIYVPW and GLHTSATNLYLH. A BLAST [47] analysis of these peptide sequences revealed various degrees of similarity to physiological proteins. We identified sequence homology to integrin alpha-1 (72% homology to VAARTGEIYVPW) and organic cation transporter 1 (60% homology to GLHTSATNLYLH); the significance of this homology must be further investigated.

We selected the two most abundant peptides for further validation. Primarily, the frequency of both peptides after the phage display experiment indicates their specificity for brain endothelial cells. Brain endothelial cells form a highly specialized interface which forms the basis of the BBB [48]. Prediction of BBB permeability has implications for the development of new CNS drugs. A small set of parameters characterizes the physio-chemical elements needed for BBB permeability. These include molecular weight, lipophilicity, hydrogen bond donors, topological polar surface area (TPSA), and ionization of compound (pKa). Overall, CNS drugs are more lipophilic, have fewer hydrogen bonds, lower TPSA, and are smaller [49]. Lipophilicity was previously identified as the most important predictor of the BBB permeability for peptides [50]. However, due to the extreme variability in amino acid sequences and the possible involvement of active transport mechanisms, validated in vitro BBB models or in vivo studies are the most valuable approaches for assessment of permeability of peptides through the BBB.

We assessed peptide permeability using the in vitro BBB model. We selected the primary rat in vitro BBB model based on a co-culture of primary rat endothelial cells with primary rat glial cells (microglia and astrocytes). It is one of the most accepted and validated BBB models which mimics passive diffusion properties and active transport mechanisms. We quantified transported peptides in the acceptor compartment by UHPLC–MS/MS. Similar to a previous study, we synthesized N-C reversed sequence peptides, WPVYIEGTRAAV and HLYLNTASTHLG, as negative controls [51]. We found that the amount of peptide increased with time and the permeability coefficients obtained for VAARTGEIYVPW peptide was 1.5 × 10^−6^ cm/s and for GLHTSATNLYLH peptide was 3.3 × 10^−7^ cm/s, respectively. The permeability experiments also showed that the control peptides had lower permeability coefficients. The peptides were transported across the in vitro BBB model two times more efficiently than control peptides. The results from permeability experiments and mass spectrometry analysis showed that according to the Pe coefficient, the VAARTGEIYVPW peptide crossed the BBB with higher efficiency than GLHTSATNLYLH. The permeability obtained for the VAARTGEIYVPW peptide was on the same order of magnitude as other known BBB shuttles such as MiniAp4 with a value of 6.7 × 10^−6^ cm/s (human primary cells) and 1.49 × 10^−6^ cm/s (bovine primary cells) [52], PhPro4 with a value of 6.88 × 10^−6^ cm/s, and NMePhe4 with a value of 6.8 × 10^−6^ cm/s—values from a parallel artificial membrane permeability assay (PAMPA) assay [33]. However, because of different primary cell culture models or cell-free systems used in these studied, the exact comparison of our peptides to these shuttles remains to be determined. To shed light on the mechanism by which the identified peptides were transported across BBB, we performed an internalization assay using primary rat endothelial cells and the SH-SY5Y neuroblastoma cell line. The biotin-labeled peptides were incubated with endothelial cells and neuroblastomas, and the process of internalization was evaluated by confocal microscopy. The results showed that both peptides were effectively internalized by primary endothelial cells, and there was no internalization or binding to non-endothelial cells. For this analysis, we also compared the transport of peptides at 37 °C and at 4 °C. Low temperature reduces cell metabolism which slows down endocytosis. The results indicated that both peptides were transported across the BBB by a transcellular mechanism.

In conclusion, we identified two BBB permeable peptides by the in vitro phage display approach. Identified peptides were internalized by endothelial cells and transported across the BBB. Our results showed that these peptides could be used to improve the penetration of therapeutics into the brain.

## 4. Materials and Methods

### 4.1. Cultivation of SH-SY5Y Neuroblastoma Cell Line

The SH-SY5Y cells (European Collection of Authenticated Cell Cultures, Sigma–Aldrich, St. Louis, MO, USA) were cultivated in MEM-F12 medium (PAA Laboratories GmbH, Germany) containing 10% fetal calf serum (FCS, Invitrogen, Carlsbad, CA), 2 mM Ultraglutamine (LONZA, Basel, Switzerland), and at 37 °C and 5% CO_2_. The medium was changed twice a week.

### 4.2. Isolation and cultivation of rat primary glial culture

Rat-mixed glial culture was prepared according to McCarthy and de Vellis [3] from cerebral cortices of 0–2 day old Sprague–Dawley rats. The cerebral cortices were dissected, stripped of the meninges, and mechanically dissociated by repeated pipetting followed by passage through a 20 µm nylon mesh. Cells were plated on 6 well plates pre-coated with poly-l-lysine (10 µg/mL, Sigma–Aldrich, St. Louis, MO) and cultivated in DMEM medium containing 10% FCS and 2 mM Ultraglutamine (LONZA, Basel, Switzerland) at 37 °C, 5% CO_2_ in a water-saturated atmosphere. For further experiments, three-week-old glial cultures were used.

### 4.3. Isolation and Cultivation of Primary Rat Brain Endothelial Cells: Development of an in Vitro BBB Model

Isolation of primary rat brain endothelial cells was done according to Watson et al. [2]. Briefly, 4 Sprague–Dawley rats (200–250 g, 6 months old) were euthanized, and the whole brains were removed. Under sterile conditions, the brainstem and cerebellum were dissected, and the midbrain, white matter, and choroid plexus were removed. The remaining cortical tissue was cleaned from meninges on dry Whatman paper. The tissue was homogenized on ice in DMEM-F12 (PAA laboratories GmbH, Germany). The brain homogenate was centrifuged at 800× *g* for 10 min at 4 °C. The supernatant was aspirated, and the pellet was resuspended in pre-warmed digestion mix containing collagenase/dispase (Roche Diagnostics, Indianapolis, IN, USA) and DNase I (Sigma–Aldrich, St. Louis, MO, USA). The tissue was incubated with a prepared digestion mix at 37 °C for 30 min with gentle shaking. Digested tissue was centrifuged at 800× *g* for 10 min at 4 °C, and the pellet was resuspended in 20% bovine serum albumin (BSA, Sigma–Aldrich, St. Louis, MO). The tissue was centrifuged at 1500× *g* for 15 min at 4 °C to obtain pellet containing microvessels with a fraction of myelin and BSA on the top, which was centrifuged again. The microvessels were pooled and resuspended in pre-warmed digestion mix and digested for 15 min at 37 °C. The microvessels pellet was again centrifuged at 800× *g* for 10 min at 4 °C and washed with culture medium containing the serum. The microvessels were cultured in DMEM-F12 medium (PAA Laboratories GmbH, Germany) containing 15% plasma-derived serum (PDS) (First Link, UK), 2 mM L-glutamine (GE Healthcare, UK), BME vitamins (Sigma–Aldrich, St. Louis, MO), heparin (Sigma–Aldrich, St. Louis, MO) and 3 µM puromycine (Sigma–Aldrich, St. Louis, MO). After 7 days, cells were seeded on the upper chamber (apical compartment) of Transwell insert with a 0.4 µm pore size (Becton Dickinson, New Jersey, USA) pre-coated with 10 µg/cm^2^ collagen type IV (Sigma–Aldrich, St. Louis, MO) and 5 µg/cm^2^ fibronectin (Sigma–Aldrich, St. Louis, MO). Endothelial cells were cultivated together with mixed glial cells cultivated as an adherent monolayer on the basolateral compartment of 12 well Cortar plates for further seven days in EBM-2 medium containing 15% plasma-derived serum (PDS), 2 mM L-glutamine, BME vitamins, and BulletKit SingleQuots (Lonza, UK). After seven days, the inserts were ready for experiments.

### 4.4. Media and Solutions

The IPTG/X-gal stock solution: 1.25 g IPTG (Sigma–Aldrich, St. Louis, MO) and 1 g X-gal in 25 mL DMSO (Sigma–Aldrich, St. Louis, MO). Tetracycline (Sigma–Aldrich, St. Louis, MO) stock suspension: 20 mg/mL tetracycline in 1 mL of 96% ethanol. LB/IPTG/X-gal plates: 1 L LB medium (AppliChem GmbH, Darmstadt, Germany), 15 g/L agar, and 1 mL IPTG/X-gal stock solution. LB/Tet plates: 1 L LB medium, 15 g/L agar and 1 mL tetracycline stock (15 mg/mL). PEG/NaCl: 20% (*w/v*) PEG 8000 (Sigma–Aldrich, St. Louis, MO), 2.5 M NaCl. Iodide buffer: 10 mM Tris-HCl (pH = 8), 1 mM EDTA and 4 M NaI (Sigma–Aldrich, St. Louis, MO).

### 4.5. Peptide Standards

All peptides and N–C reversed sequence peptides used in the study were synthesized by ThermoFischer Scientific (Waltham, MA, USA). The peptide purity was >98%.

### 4.6. Screening of Phage Display Library Using Primary Rat Brain Endothelial Cells

A phage library of random 12-mer peptides (Ph.D. TM-12 Phage Display Peptide Library) was purchased from New England Biolabs (Beverly, MA, USA). Neuroblastoma and endothelial cells were grown to a confluent monolayer in 100-cm^2^ plates, and incubated in the serum-free medium for 1 h before panning. In the first step, the phage library was applied to neuroblastoma cells to remove phages recognizing surface proteins of SH-SY5Y. Phage library of 2 × 10^11^ pfu in 2 mL of washing/blocking buffer (0.1% BSA, 0.1% Tween 20 in DMEM) was added to the neuroblastoma cells and incubated for 1 h at room temperature with agitation. After the incubation, the phages, which did not bind to the neuroblastoma cells, were transferred to the endothelial cells and again incubated for 1 h at room temperature with agitation. Unbound phages were washed away (10× with washing buffer), and endothelial cell surface-bound phages were recovered with low-pH elution buffer (100 mM glycine, pH 2.2). Eluted phages were used for amplification in *Escherichia coli* bacteria (ER2738, New England Biolabs, Beverly, MA). Amplified phages were applied for the additional three rounds of selection, similar to described above. After the fourth round, phage DNA from phages specifically bound to endothelial cells was isolated and sequenced. Phage amplification, phage titration, purification of single-stranded M13 viral DNA, and other phage display methods were performed according to manufacturer recommendation (Manual for Ph.D. TM-12 Phage Display Peptide Library, New England Biolabs).

Briefly, the phages were amplified by adding 200 µL of the eluate to 50 mL of *E. coli* cells (OD_600_ = 0.6, New England Biolabs) and then vortexed and incubated for 4 h at 37 °C with vigorous shaking. The culture was transferred to a centrifuge tube and spun for 20 min at 5000 rpm at 4 °C. The upper part of the supernatant was transferred to a fresh tube, and 18 mL of 20% PEG with 2.5 M NaCl was added. The phage was allowed to precipitate 24 h at 4 °C. The following day, the phages were precipitated by centrifugation for 30 min at 5000 rpm at 4 °C. The supernatant was discarded, and the pellet was resuspended in 1 mL of PBS and transferred to a new tube. The residual cells were discarded by centrifugation for 10 min at 20,000 rpm at 4 °C. The supernatant was recovered in a new tube. A second precipitation was performed with 200 µL of 20% PEG with 2.5 M NaCl for 1 h in ice. The phages were recovered by spinning for 30 min at 20,000 rpm at 4 °C. The supernatant was discarded, and the pellet was dissolved in 200 µL of PBS and used for the next round of bio-panning. Serial dilutions of phages were prepared in LB medium (10^6^–10^10^ fold for amplified phage culture supernatant and 10^1^–10^4^ fold for unamplified panning eluates). Ten microliters of each phage dilution were added to 200 µL of *Escherichia coli* cells and then vortexed and incubated for 5 min. The infected phages were transferred to LB/IPTG/X-gal plates. The plates were incubated overnight at 37 °C and the plaques were counted to obtain the titer in plaque-forming units (pfu):

Plaque forming units (pfu) = (number of plaques × dilution factor)/10 (µL)(1)

Bacterial cells were infected with the selected phage colony, which was picked from the titration plates, and the phages were amplified. In the final step, the phages were suspended in 100 µL of iodide buffer by vigorously tapping the tube. 500 µL of ethanol was added and incubated for 30 min at room temperature. The single-stranded phage DNA was recovered by spinning for 10 min at 20,000× *g* at 4 °C. The pellet was washed with 1 mL of 70% cold ethanol and centrifuged for 10 min at 20,000× *g* at 4 °C. The pellet was dried and resuspended in 50 µL of sterile deionized water. The peptide-encoding nucleotide sequence was determined with the −96 gIII sequencing primer included in Ph.D.-12^TM^ Phage Display Peptide Library.

### 4.7. Permeability of Endothelium to Lucifer Yellow

Transendothelial electrical resistance (TEER) measurement was used to measure the resistance of tight junctions in our BBB model. The Transwell inserts (in a 12 well format, containing an endothelial layer or without cells) were transferred into 12-well plates containing 1.5 mL of Ringer-HEPES solution (150 mM NaCl, 5.2 mM KCl, 2.2 mM CaCl_2_, 0.2 mM MgCl_2_·6H_2_O, 6 mM NaHCO_3_, 5 mM HEPES, 2.8 mM glucose; pH 7.4) per well. The cell culture medium was removed from the inserts, and 0.5 mL of Ringer-HEPES solution containing 10 μM Lucifer Yellow (LY) (Sigma–Aldrich, St. Louise, Missouri, United States) was added to the upper (apical) compartment. All incubations were performed at 37 °C. After different time points (5, 10, 15, 30, and 60 min), a 200 μL aliquot from each lower compartment was placed in a fluorimeter for quantification (excitation wavelength: 428 nm; emission wavelength: 536 nm). The endothelial permeability coefficient (Pe) of LY was calculated in cm/s. The permeability values of the inserts (PSf, for inserts with a coating only) and the insert plus endothelium (PSt, for inserts with a coating and cells) were taken into consideration by applying the following equation:

1/PSe = 1/PSt − 1/PSf(2)

To obtain the endothelial permeability coefficient (Pe, in cm/s), the permeability value (PSe) corresponding to the endothelium alone was then divided by the insert’s porous membrane surface area.

### 4.8. Immunocytochemistry

Primary endothelial cells were incubated with biotinylated peptides (final concentration 1 µM) for 1 h. Cells were washed three times with phosphate buffer PBS (137 mM NaCl, 2.7 mM KCl, 10 mM Na_2_HPO_4_, 2 mM KH_2_PO_4_, pH 7.4). Cells were fixed with pre-warmed 4% paraformaldehyde (PFA, Sigma–Aldrich, St. Louis, MO) for 10 min at 37 °C and permeabilized with 0.3% TritonX-100 for 3 min (Sigma–Aldrich, St. Louis, MO). Cells were blocked with PBS with 5% BSA and 0.1% Triton-X100 for 40 min at room temperature. Subsequently, cells were incubated for 1 h with primary antibody diluted in blocking buffer. For immunocytochemical analysis of endothelial cells, polyclonal rabbit anti-rat pan-laminin antibody was used (Sigma–Aldrich, St. Louis, MO). After the staining with the primary antibody, endothelial cells were washed three times with PBS and incubated with fluorescently labeled secondary antibody goat anti-rabbit Alexa Fluor 546 and streptavidin-Alexa 488 diluted in blocking buffer (1:3000, Invitrogen, Carlsbad, CA, USA) for 1 h at room temperature. Finally, cells were washed 3 times with PBS, dried in 96% ethanol and mounted in VECTASHIELD HardSet fluorescent mounting media (Vector Laboratories, UK). Immunofluorescent staining was analyzed by Axiovert 200 fluorescent microscope (Zeiss, Jena, Germany) and LSM 710 confocal microscope (Zeiss, Jena, Germany).

### 4.9. Quantification of Peptide Transport across in Vitro BBB by Mass Spectrometry

For each peptide, three 200 µL aliquots of luminal and abluminal compartments were mixed with 200 µL of acetonitrile containing 0.2% formic acid (Sigma–Aldrich, St. Louis, MO). The calibration curve for each peptide was prepared in Ringer Hepes/acetonitrile (50:50) with 0.1% formic acid. Ten calibration points ranging from 0.1 ng/mL to 200 ng/mL were used. Quantitative analysis of peptides was carried out by ultra-performance liquid chromatography-tandem mass spectrometry using ACQUITY ultra-performance liquid chromatography (UPLC) H-class system (Waters Corporation, Milford, MA) coupled to a triple-quadrupole mass spectrometer Xevo TQD (Waters Corporation, Milford, MA). Chromatographic separation was performed in reversed-phase mode on ACQUITY UPLC BEH C8 1.7 µm (2.1 × 50 mm) column (Waters, Prague, Czech Republic) maintained at 35 °C. The flow rate was 0.5 mL/min^−1^. Mobile phase A consisted of 0.1% formic acid in water, and mobile phase B consisted of 100% acetonitrile. The following elution gradient was used: 5% B in 0–0.5 min, 5–50% B in 0.5–1.7 min, 50–90% B in 1.7–1.8 min, 90% B in 1.8–3.0 min, 90–5% B in 3.0–3.3 min, 5% B in 3.3–3.5 min. The injection volume was 5 µL. The mass spectrometer was operated in MRM mode. Samples were analyzed in positive electrospray mode; the capillary voltage was 2.00 kV. The source and desolvation temperatures were 150 °C and 450 °C, respectively, and the flow rate of desolvation gas was 700 L/h. Acquisition and evaluation of acquired data were carried out using MassLynx 4.1. software (Waters, Prague, Czech Republic).

### 4.10. High-Resolution Mass Spectrometry Analysis of Peptides

The high-resolution mass spectrometry analysis was performed using a Synapt-G2Si instrument coupled to the Acquity M-class nano-LC system (Waters, Prague, Czech Republic). The HSS T3 column (100 Å, 1.8 µm, 75µm × 250 mm) was used for peptide separation. The column was heated to 40°C. Mobile phase A was composed of 0.1% formic acid in deionized water (*v/v*), and mobile phase B consisted of acetonitrile with 0.1% formic acid (*v/v*). The mobile phase gradient program was as follows: 2% B in 0–5 min, 2–40% B in 5–35 min and then 2% B in 35–50 min. The flow rate was 0.3 µL min^−1^, and the injection volume was 1 µL (final concentration 100 fmol). Data were acquired with MassLynx 4.2 software and analyzed by UNIFI Scientific Information System (Waters, Prague, Czech Republic).

### 4.11. Toxicity Analysis (Cell Viability Measurement)

The toxicity of the peptides was measured by ToxiLight^TM^ Non-Destructive Cytotoxicity BioAssay Kit (Lonza). The ToxiLight^TM^ BioAssay Kit is a bioluminescent, non-destructive cytolysis assay kit designed to measure the release of the enzyme adenylate kinase (AK) from damaged cells. We mixed 100 µL of a sample and 50 µL of lysis reagent and incubated it for 10 min at room temperature. The resulting luminescence was measured using a Fluoroscan Ascent FL (MTX Lab Systems, Inc., Bradenton, FL, USA).

## Figures and Tables

**Table 1:** Identified peptides recognizing endothelial cells.

Phage Clone	Sequence	Frequency
1.1	IGVRGCIWDPQP	1/35
1.2	**VAARTGEIYVPW**	8/35
1.3	**GLHTSATNLYLH**	11/35
1.4	HAEHSQVRGAAN	1/35
1.5	AYPQKFNNNFMS	1/35
1.6	VIGPLDRHAHLK	2/35
1.7	APTAYNKNDWAL	1/35
1.8	NRPDSAQFWLHH	1/35
1.9	IDLRPKDDLPQP	2/35
1.10	IEASFYDAPRGG	1/35
1.11	GSWGLNDSSAAY	2/35
1.12	HASGSISGFWPN	1/35
1.13	VNMVPIGGNQVV	1/35
1.14	LNTNSQLQTNNA	2/35

Note—peptides selected for further experiments are labeled in bold.

**Table 2:** Selected reaction monitoring (SRM) conditions of peptides.

Compound Name	Precursor Ion (*m/z*)	Product Ion (*m/z*)	Dwell Time (s)	Cone Voltage (V)	Collision Energy (eV)
WPVYIEGTRAAV	681.7	302.2	0.03	25	20
	681.7	1061.7	0.02	25	20
GLHTSATNLYLH	521.16	464.8	0.03	30	30
	521.16	831.6	0.02	30	30
VAARTGEIYVPW	681.45	703.21	0.063	25	20
	681.45	979.36	0.063	25	20
HLYLNTASTHLG	521.32	229.11	0.03	25	15
	521.32	813.35	0.03	25	15

## Supplementary Materials

The following are available online, Figure S1: Cytotoxicity of peptides, Figure S2: Internalization of peptides into SH-SY5Y neuroblastoma cells, Table S1: Physico-chemical properties of peptides.
