# Supplementary material for: Novel Blood–Brain Barrier Shuttle Peptides Discovered through the Phage Display Method
Source: Molecules. 2020 Feb 17;25(4):874. doi: 10.3390/molecules25040874 (PMC7070575; doi:10.3390/molecules25040874)
Supplement: Supplementary file 1 [file molecules-25-00874-s001.pdf]

Supplementary Materials

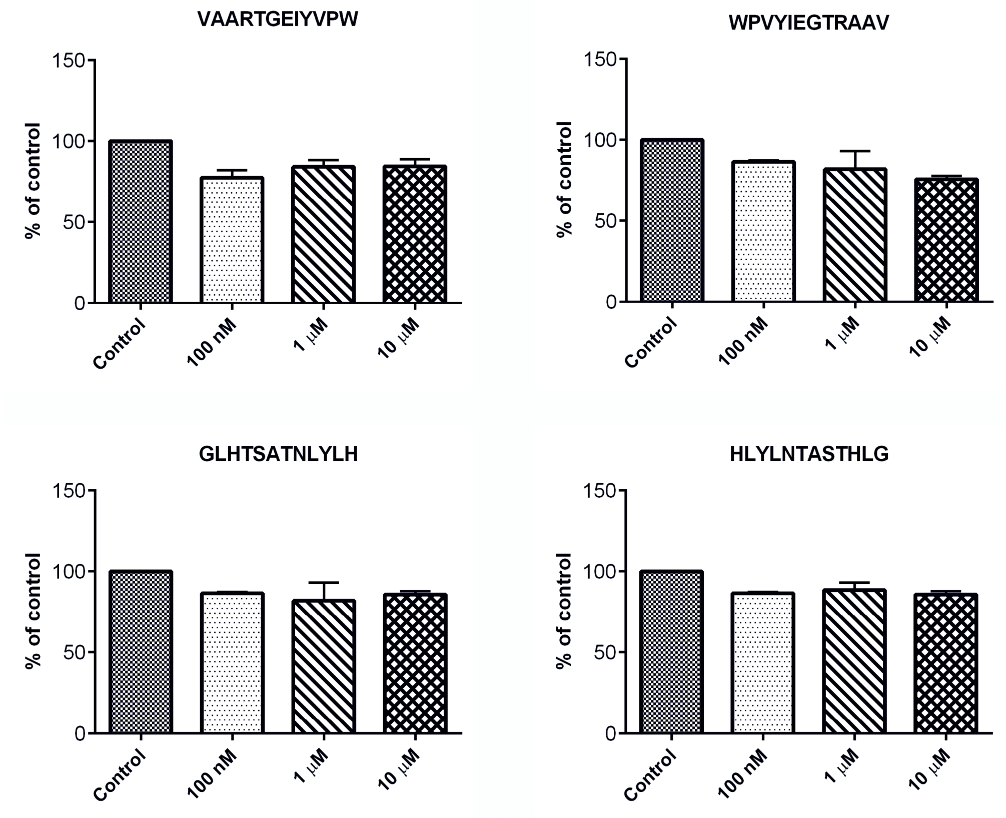

Figure S1. Cytotoxicity of peptides. The values represent the mean ±SEM of two independent experiments conducted in sextuplicates.

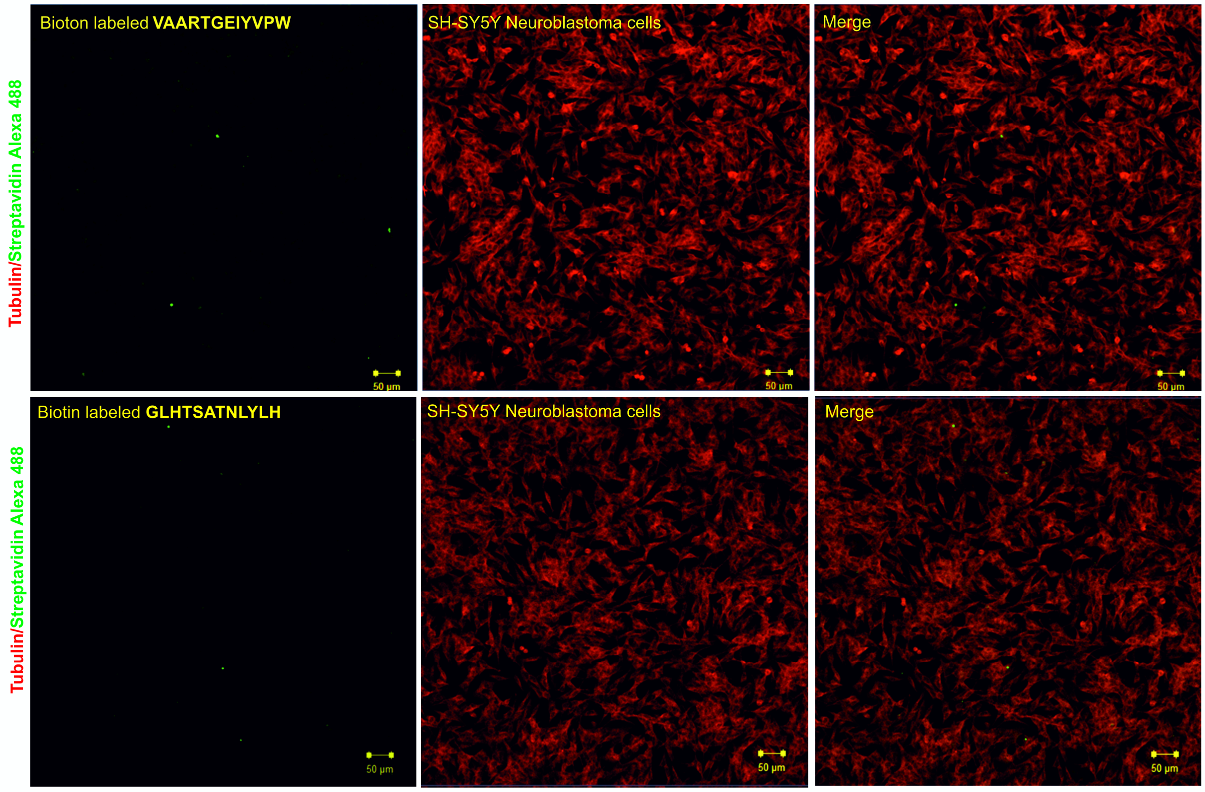

Figure S2. Internalization of peptides into SH-SY5Y neuroblastoma cells. Confocal microscopy of neuroblastoma SH-SY5Y cells after 1 hour of co-incubation with VAARTGEIYVPW and GLHTSATNLYLH peptides. Peptides are shown in green, tubulin in red. Scale bar 50 µm.

Table S1. Physico-chemical properties of peptides.

Phage clone	Sequence	Frequency	Molecular weight (g/mol)	Hydrophobic (%)	Acidic (%)	Basic (%)	Neutral (%)	Extinction coefficient ($M^{-1}cm^{-1}$)	Net charge (pH 7)	TPSA	LogP	LogS	pKa
1.1	IGVRGCIWDPQP	1/35	1340.55	50	8.33	8.33	33.33	5690	-0.1	523.91	-5.05	-7.64	7.35
**1.2**	**VAARTGEIYVPW**	**8/35**	**1361.54**	**58.33**	**8.33**	**8.33**	**25**	**6970**	**0**	**526.32**	**-2.94**	**-8.81**	**7.37**
**1.3**	**GLHTSATNLYLH**	**11/35**	**1326.46**	**33.33**	**0**	**16.67**	**50**	**1280**	**0.2**	**556.21**	**-5.88**	**-6.22**	**7.23**
1.4	HAEHSQVRGAAN	1/35	1276.32	33.33	8.33	25	33.33	0	0.2	646.38	-6.22	-3.17	7.09
1.5	AYPQKFNNNFMS	1/35	1460.61	41.67	0	8.33	50	1280	1	613.47	-5.42	-6.01	7.33
1.6	VIGPLDRHAHLK	2/35	1355.59	50	8.33	33.33	8.33	0	1.2	557.22	-5.42	-8.11	7.39
1.7	APTAYNKNDWAL	1/35	1363.47	50	8.33	8.33	33.33	6970	0	580.37	-5.35	-6.21	7.39
1.8	NRPDSAQFWLHH	1/35	1507.61	41.67	8.33	25	25	5690	0.2	653.38	-5.75	-8.31	5.93
1.9	IDLRPKDDLPQP	2/35	1406.58	50	25	16.67	8.33	0	-1	600	-5.80	-6.35	7.37
1.10	IEASFYDAPRGG	1/35	1282.36	41.67	16.67	8.33	33.33	1280	-1	552.57	-5.56	-5.98	7.20
1.11	GSWGLNDSSAAY	2/35	1227.24	33.33	8.33	0	58.33	6970	-1	560.50	-5.72	-3.68	7.12
1.12	HASGSISGFWPN	1/35	1259.33	41.67	0	8.33	50	5690	0.1	522.86	-5.45	-4.90	7.09
1.13	VNMVPIGGNQVV	1/35	1226.45	58.33	0	0	41.67	0	0	503.89	-4.53	-4.25	7.23
1.14	LNTNSQLQTNNA	2/35	1317.36	25	0	0	75	0	0	702.65	-6.09	-0.09	7.23
